# Growing in Mixed Stands Increased Leaf Photosynthesis and Physiological Stress Resistance in Moso Bamboo and Mature Chinese Fir Plantations

**DOI:** 10.3389/fpls.2021.649204

**Published:** 2021-05-20

**Authors:** Chunju Peng, Yandong Song, Chong Li, Tingting Mei, Zhili Wu, Yongjun Shi, Yufeng Zhou, Guomo Zhou

**Affiliations:** ^1^State Key Laboratory of Subtropical Silviculture, Zhejiang A&F University, Lin’an, China; ^2^Zhejiang Provincial Collaborative Innovation Center for Bamboo Resources and High-efficiency Utilization, Zhejiang A&F University, Lin’an, China; ^3^Key Laboratory of Carbon Cycling in Forest Ecosystems and Carbon Sequestration of Zhejiang Province, Zhejiang A&F University, Lin’an, China; ^4^School of Environmental and Resources Science, Zhejiang A&F University, Lin’an, China; ^5^Lishui Academy of Agricultural and Forestry Sciences, Lishui, China

**Keywords:** leaf-level gas exchange, stand carbon sequestration, chlorophyll fluorescence, antioxidant enzyme, water use efficiency, bamboo-fir mixture

## Abstract

Mixed-stand plantations are not always as beneficial for timber production and carbon sequestration as monoculture plantations. Systematic analyses of mixed-stand forests as potential ideal plantations must consider the physiological-ecological performance of these plantations. This study aimed to determine whether mixed moso bamboo (*Phyllostachys pubescens* (Pradelle) Mazel ex J. Houz.) and Chinese fir (*Cunninghamia lanceolata* (Lamb.) Hook.) stands exhibited better physiological-ecological performance than monoculture plantations of these species. We analyzed leaf photosynthesis, chlorophyll fluorescence, antioxidant enzyme activities, chlorophyll content and leaf chemistry in a moso bamboo stand, a Chinese fir stand and a mixed stand with both species. The results showed that both species in the mixed stand exhibited significantly higher leaf net photosynthesis rate (Amax), instantaneous carboxylation efficiency (CUE), chlorophyll content, maximum quantum yield of photosynthesis (Fv/Fm), photochemical quenching coefficient (qP), PSII quantum yield [Y(II)], leaf nitrogen content, and antioxidant enzyme activities than those in the monoculture plantations. However, the non-photochemical quenching (NPQ) in Chinese fir and 2-year-old moso bamboo was significantly lower in the mixed stand than in the monocultures. In addition, the water use efficiency (WUE) of Chinese fir was significantly higher in the mixed stand. The results suggest that the increase in leaf net photosynthetic capacity and the improved growth in the mixed stand could be attributed primarily to the (i) more competitive strategies for soil water use, (ii) stronger antioxidant systems, and (iii) higher leaf total nitrogen and chlorophyll contents in the plants. These findings suggest that mixed growth has beneficial effects on the leaf photosynthesis capacity and physiological resistance of moso bamboo and Chinese fir.

## Introduction

Moso bamboo (*Phyllostachys pubescens* (Pradelle) Mazel ex J. Houz.) is extensively distributed in southeastern and southern Asia and is well known for its rapid growth and important ecological and economic benefits (Guan et al., 2015; Zhang et al., 2015). For example, moso bamboo plants can grow in height by up to 1 m per day during the period of their fastest growth (Ueda, 1960) and complete their vertical growth within 35–40 days after shoots emerge from the soil in the spring (Song et al., 2016). Due to its rapid growth and substantial ecological benefits and carbon sink functions, moso bamboo is grown in monocultures under intensive management. Moso bamboo forests are estimated to cover an area of 4.43 million ha, which represents 80% of their global distribution and 63% of the bamboo forests in China (Li et al., 2013; Song et al., 2015; Tang et al., 2017). However, long-term monoculture planting under intensive management has resulted in the loss of species biodiversity, declining soil fertility and poor ecological stability (Wang et al., 2016). Low timber prices and increased labor costs have also exacerbated lax management practices and even the abandonment of moso bamboo plantations in China and Japan in recent years (Shinohara et al., 2019). The vigorous rhizome system of moso bamboo, which spreads laterally underground, can rapidly expand and invade adjacent forests (Shinohara et al., 2019). Therefore, moso bamboo often impedes the development of adjacent secondary broad-leaved and/or coniferous plantations (Wang et al., 2016; Song et al., 2017). Moso bamboo has similar environmental and site requirements for growth as Chinese fir (*Cunninghamia lanceolata* (Lamb.) Hook.), and the distributions of their plantations are often similar; therefore, monoculture plantations can be gradually transformed into mixed forests via the interaction of roots and litter in the transition zone (Song et al., 2014; Tu et al., 2014).

Chinese fir is one of the most important plantation trees due to its fast growth, straight and decay-resistant timber, and high economic value (SFAPRC, 2015; Wang et al., 2018). It has been widely planted in China, with an area of 8.95 million hectares that accounts for 19% of the planted forest area in China and 6.1% of the global planted forest area (Yang et al., 2018). Chinese fir plantations are the major forest carbon sinks in China due to their high carbon sequestration potential (Wang et al., 2018). Similar to that of other monocultures, the sustainability of Chinese fir plantations is threatened by biodiversity reduction, production losses and declining soil fertility (Chen et al., 2014; Wu et al., 2014; Liu et al., 2018). Thus, forest management practices usually convert monocultures to mixed stands to maintain an ecological balance (Liu et al., 2018), increase stand-level productivity (Li et al., 2015; Huang et al., 2018), increase individual tree growth rates (Andrés et al., 2017), implement environmental restoration, and increase soil fertility (Liu et al., 2018).

Mixed plantations have been reported to have a higher carbon sequestration capacity than monocultures (Liang et al., 2016; Huang et al., 2018; Zhang et al., 2019). For example, 15 years after reforestation, the carbon stocks in mixed Chinese fir and *Alnus cremastogyne* Burk. plantations were 11% higher than those in pure Chinese fir plantations (Wang et al., 2009). Similarly, the conversion of a pure Scots pine (*Pinus sylvestris* L.) plantation into a mixed stand of Scots pine and European beech (*Fagus sylvatica* L.) resulted in higher carbon sequestration (Pretzsch et al., 2016). Huang et al. (2018) also found positive effects of tree species richness on stand-level productivity. These results suggest that mixed plantations are a better strategy than monocultures for maintaining species diversity and community stability to cope with forest degradation. The higher stand-level production in mixed plantations can be explained by enhanced structural heterogeneity (Pretzsch et al., 2016), species richness a mechanism (Huang et al., 2018), the competitive production principle (Kelty, 2006) and increases in photosynthesis and water-use efficiency (Forrester et al., 2012). However, mixed planting does not always benefit productivity, depending on the strength of both intra- and interspecific competition (Forrester, 2015; Forrester and Bauhus, 2016). Therefore, each species combination involved should be considered when studying the performance of a mixed stand (Andrés et al., 2017). Increasing photosynthesis has been shown to increase biomass and carbon sequestration in a range of species (Forrester et al., 2012). Previous studies on mixed moso bamboo forests have focused mainly on broad-leaved forests and their production (Bai et al., 2016; Shinohara et al., 2019), while less information is available on mixed-species forests comprising moso bamboo and coniferous trees.

Photosynthesis, which converts light energy into usable chemical energy for various metabolic processes, is the most crucial process for plant growth and development (Makino, 2011). It is also a complex physiological process that is very sensitive to changes in the environment (Ashraf and Harris, 2013). External environmental factors cannot only directly affect plant photosynthesis but can also indirectly affect photosynthesis by affecting the physiological conditions inside the plants (Cano et al., 2014). Reactive oxygen species (ROS) are often produced during photosynthesis in chloroplasts (Wu et al., 2018b). Increasing the activity of major ROS scavenging enzymes can increase the protection of plant photosynthetic capacity (Payton et al., 2001). Nitrogen is involved mainly in light energy capture, electron transfer and carboxylation processes in photosynthesis, so the nitrogen content of leaves is closely related to photosynthesis (Wu et al., 2018b). As the most critical photosynthetic pigment in plants, chlorophyll has an important influence on the plant photosynthetic rate, primary productivity and growth (Forrester et al., 2012). Its synthesis process and content are related to the site conditions. However, our understanding of the physiological-ecological responses of mixed-species stands of moso bamboo and Chinese fir also lags well behind our understanding of the responses of single-species stands (Guan et al., 2015; Shinohara et al., 2019).

In this context, the present study sought to quantify the net photosynthetic capacity of moso bamboo and Chinese fir in mixed forests and the effect of mixed planting on the growth of these two species. We investigated how the photosynthetic parameters, fluorescence parameters, enzyme activity and leaf chemistry of both species responded to growing in mixed stands. The specific objectives of this study are to determine whether the mixed planting would increase the photosynthetic capacity of the plants and improve their physiological stress resistance. The study of moso bamboo and Chinese fir in a mixed stand will contribute to understanding current patterns in tree growth, implementing efficient plantation management strategies and achieving higher economic returns.

## Materials and Methods

### Study Site

**FIGURE 1:**
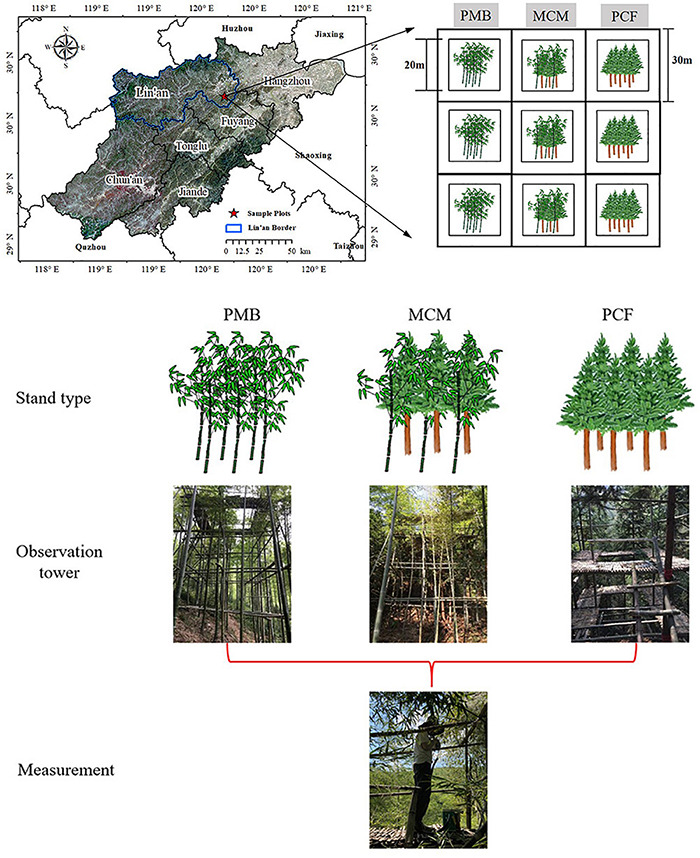
Location of the sample site and sampling design. The white area indicates a buffer; 20 m refers to the length of the border around the plots. See section “Materials and methods” for details.

The study site was located in Banqiao town, Lin’an city (30°10’ N, 119°45’ E), Zhejiang Province (Figure 1), China. The mean annual precipitation at the study site is 1,420 mm, and the monthly precipitation is 118 mm, ranging from 47 mm in December to 231 mm in June. The mean annual temperature is 17.49°C, with monthly mean temperatures reaching a maximum of 24°C in July and a minimum of 3°C in January. The area receives an average of 1,774 h of sunshine per year and 236 h of sunshine per month, ranging from 108 h per month in February to 216 h per month in August. The soil is a slightly acidic red soil derived from siltstone (Li et al., 2018). The original Chinese fir plantation at the study site was all logged approximately 15 years ago, but Chinese fir trees have continued to grow on the plantation. Thus, all Chinese fir plantation trees were 15 years old in the study site. Furthermore, a plantation of moso bamboo was established adjacent to the Chinese fir plantation at approximately the same time as the Chinese fir plantation was logged. The transition zone between the Chinese fir and moso bamboo plantations is approximately 30–50 m in width. In this zone, the moso bamboos reach the same height as the canopy formed by the Chinese fir. Moso bamboos more than 4 years old are usually harvested in November during even-numbered years to maximize the economic benefits. Thus, the moso bamboo plantations are uneven-aged forests with a 2-year difference in the age of the trees (Song et al., 2016).

### Experimental Design

In February 2019, three experimental sites were established, including a monoculture moso bamboo plantation site (PMB), a monoculture Chinese fir plantation site (PCF) and a mixed forest site with both species (MCM). There were three replicate plots in each site, meaning that a total of 9 plots (20 m × 20 m) were established (Figure 1). The plots were separated by buffer zones of at least 20 m to keep them independent of each other. The stand characteristics are summarized in Table 1.

**TABLE 1:** Stand and average tree characteristics in moso bamboo and Chinese fir monocultures and a mixed stand.

**Stand and tree type**	**Mean DBH (cm)**	**Mean height (m)**	**Density (stems ha^–1^)**	**Biomass (t ha^–1^)**
Monoculture, Chinese fir	10.9 ± 3.6	7.2 ± 2.4	2366.7 ± 208.2	37.3 ± 11.5
Mixed, Chinese fir	8.2 ± 2.2	7.8 ± 2.1	2405.6 ± 980.9	24.6 ± 7.2
Mixed, Moso bamboo	7.6 ± 1.7	9.1 ± 2.0	1649.1 ± 392.8	16.7 ± 1.2
Monoculture, Moso bamboo	9.6 ± 1.7	8.9 ± 1.5	3051.9 ± 168.3	29.8 ± 2.0

### Leaf-Gas Exchange Measurements, Chlorophyll a Fluorescence and Chlorophyll Content Determination

A 20 m tall observation tower with a walkway was built to reach the crown of the trees in each plot of the PCF, PMB, and MCM sites (9 observation towers in total). Around each tower, three Chinese fir trees were selected in the PCF site, six moso bamboo plants (three 1-year-old and three 2-year-old moso bamboo plants) were selected in the PMB site, and a total of nine plants (three Chinese fir trees, three 1-year-old and three 2-year-old moso bamboo plants) were selected in the MCM site. Then, several mature and healthy leaves of both Chinese fir and moso bamboo trees on the southern side from the middle layer canopy of the selected sample plants in each plot were chosen randomly for measurement. These leaves were similar in size and were all taken from the middle of each twig (Van Goethem et al., 2013; Zhang et al., 2017a; Wang et al., 2019; Figure 1). The leaf gas exchange and fluorescence parameters were measured, chlorophyll contents were determined and samples were collected in the tower in Spring (May) 2019.

The net photosynthesis rate (Amax), stomatal conductance (gsw), intracellular CO_2_ concentration (Ci), and transpiration rate (Tr) were measured with an open gas-exchange system connected to a leaf fluorometer chamber (2 cm^2^; Li-6800; Li-Cor, Inc., United States). Measurements from the selected leaves were taken on sunny days between 8:00 and 16:00. The CO_2_ concentration was maintained at 400 μmol mol^–1^ at a leaf temperature of 25°C, and the vapor pressure deficit was maintained as close to 1 kPa as possible. Several leaves (two moso bamboo or six to eight Chinese fir leaves) were flat into the leaf chamber and with full covered, when measuring photosynthesis. Once a steady state was reached (usually 10 min after clamping the leaf), data were recorded every 5 s for a total of 10 data points for every single leaf. The WUE was calculated as WUE = Amax/Tr, and the instantaneous carboxylation efficiency (CUE) was calculated as CUE = Amax/Ci.

The fluorescence parameters were measured with a PAM-2500 instrument (WALZ Inc., Germany). The leaves (one moso bamboo of three to four Chinese fir leaves) were fully dark adapted with leaf clips for approximately 20 min and then exposed to weak actinic light (0.05 μmol m^–2^ s^–1^) to detect the initial minimal fluorescence (Fo). Then, a saturating light pulse (6,000 μmol m^–2^ s^–1^) was applied for 2 s to detect the maximal fluorescence (Fm). The maximal potential quantum yield of photosystem PSII was calculated as Fv/Fm = (Fm-Fo)/Fm. Leaves were exposed to the saturating light pulse repeatedly to determine the non-photochemical quenching (NPQ), photochemical quenching coefficient (qP) (Genty et al., 1989) and effective quantum yield of PSII [Y(II)] (Murchie and Lawson, 2013). A detailed documentation of the measurement of the fluorescence parameters is given by Murchie and Lawson (2013). The chlorophyll content was measured with a SPAD-502 portable chlorophyll meter (Minolta Camera Co., Ltd., Osaka, Japan). Ten mature and healthy leaves per selected sample plants from the middle layer canopy in each plot were chosen randomly for chlorophyll content measurement. For each leaf, the mean of three readings was used to represent the chlorophyll content.

### Extraction and Enzyme Activity Assay

After the in situ parameters were measured, the measured leaves were collected in plastic bags and immediately transported to the laboratory for further measurements. Fresh leaf samples (0.5 g) were collected for extraction. The enzymes were extracted at 4°C with 5 mL of 50 mM potassium phosphate buffer (pH 7.8). The extracting solutions were centrifuged at 8,000 × g at 4°C for 15 min, and the homogenate was stored at 4°C for the analysis of superoxide dismutase (SOD), peroxidase (POD), catalase (CAT), and Rubisco activity.

SOD activity was measured according to the method of Giannopolitis and Ries (1977), where the activity was determined by inhibiting the effect of *β*-nitro blue tetrazolium chloride (NBT) on photoreduction. POD and CAT activity was determined by the method of Chance and Maehly (1955). The reaction was conducted at 470 and 240 nm, and extinction coefficients of 2.47 and 36 Mm cm^–1^ were used to determine the activity of POD and CAT, respectively. Rubisco was quantified by capillary electrophoresis using a method modified from Warren et al. (2000).

The malondialdehyde (MDA) content was determined with the thiobarbituric acid method (TBA) (Li and Wang, 2003). Leaf tissue samples of 0.5 g were homogenized in 2 mL supernatant and a 2-mL mixture of TBA (0.6%, v/v) and trichloroacetic acid (TCA, 10%, v/v). The mixture was heated for 25 min at 100°C. Then, the mixture was centrifuged at 5,000 × g for 20 min after cooling. The supernatant was analyzed at 532, 600, and 450 nm with a spectrophotometer. The MDA content was calculated using the following formula: C (μM) = 6.45 (OD_532_ - OD_600_) - 0.56OD_450_.

### N Content Analysis

Nutrient contents were determined in leaf samples oven dried at 105°C during the first 30 min, then at 65°C until reaching a constant weight and then ground with a grinder (DFT-50A, Wenling LINDA Machinery Co., Ltd., China). Total leaf nitrogen content was determined using a Sumigraph NC-80 high-sensitivity CN analyser (Sumitomo Chemical Industry Co., Ltd., Japan).

### Statistical Analyses

To determine the effects of mixture, species (Chinese fir and two ages of moso bamboo) and their interaction on the photosynthetic, chlorophyll a parameters, enzyme activity and leaf chemistry parameters, data were subjected to a two-way ANOVA. Similarly, to determine the effects of mixture, age and their interaction effects on the photosynthetic, chlorophyll a parameters, enzyme activity and leaf chemistry parameters in moso bamboo, data were subjected to a two-way ANOVA. One-way ANOVA was used to analyze the effect of mixture on each parameter. Before the ANOVAs, the data were checked for normality and homogeneity of variances and log-transformed to correct deviations from these assumptions as needed. *Post hoc* comparisons were tested using the least significant difference (LSD) test at a significance level of *P* < 0.05. The changes in on photosynthetic performance parameters (Amax, WUE, CUE, Fv/Fm, NPQ, and chlorophyll content) was conducted with a fixed-effects model (Forrester and Albrecht, 2014; Andrés et al., 2017). We included as fixed factors “mixture,” “age,” and “species” to detect the differences in the rates of change of 1-year-old moso bamboo, 2-year-old moso bamboo and Chinese fir of photosynthetic performance parameters. All of the data were analyzed using SPSS statistical software (version 19.0, Armonk, NY, United States). Raincloud plots were used to evaluate the distributions of the average of various photosynthetic parameters for both species in the monoculture plantation and mixed stand. In addition, radar charts were drawn using Origin 2021 to estimate the average fluorescence parameters of the leaves of both species in the monocultures and mixed stand. The radial column chart was drawn using the ggplot2 package in R statistical software to estimate the average antioxidant enzyme activities of the leaves of both species in the monocultures and mixed stand (R v3.6.1).

## Results

The results of the two-way ANOVA suggested that mixture, species and their interaction had significant effect on WUE and CUE (*P* < 0.001; Table 2). Similarly, the results also suggested that mixture, age and their interaction effect had significant effect on WUE and CUE in moso bamboo (*P* < 0.001; Table 3). In addition, the mixture had no significant effect on NPQ (ns; Table 3), while age and the interaction of mixture and age had significant effect on NPQ (*P* < 0.001; Table 3).

**TABLE 2:** Results of two-way ANOVA on the effects of mixture and species on photosynthetic performance parameters.

**Source of variation/factors**	**Mixture**		**Species**		**Interaction**	
	***F*-value**	**Sig**	***F*-value**	**Sig**	***F*-value**	**Sig**
Amax	5.105	*	0.83	0.368	0.073	0.078
WUE	126.267	***	285.84	***	230.142	***
CUE	36.764	***	16.96	***	7.134	**
Fv/Fm	14.568	***	113.48	***	0.070	0.792
NPQ	0.857	0.359	5.37	*	3.613	0.063
Chlorophyll content	3.167	0.081	330.02	***	2.662	0.109

*p < 0.05; **p < 0.01; ***p < 0.001.

**TABLE 3:** Results of two-way ANOVA on the effects of mixture and age on photosynthetic performance parameters.

**Source of variation/factors**	**Mixture**		**Age**		**Interaction**	
	***F*-value**	**Sig**	***F*-value**	**Sig**	***F*-value**	**Sig**
Amax	33.787	***	299.612	***	3.157	0.085
WUE	60.987	***	119.218	***	43.299	***
CUE	62.859	***	290.444	***	18.308	***
Fv/Fm	36.297	***	87.115	***	1.881	0.180
NPQ	1.454	0.237	16.448	***	33.597	***
Chlorophyll content	0.033	0.857	0.999	0.325	1.259	0.270

***p < 0.001.

Using a fixed-effects model, we also found significant effects of mixture on CUE and NPQ of 1-year-old moso bamboo and Chinese fir (*P* < 0.001; Supplementary Table 1). In addition, mixture has a negative effect on WUE of 1-year-old moso bamboo, while the positive effect on WUE of Chinese fir (*P* < 0.01; Supplementary Table 1).

### Photosynthetic Parameters

**FIGURE 2:**
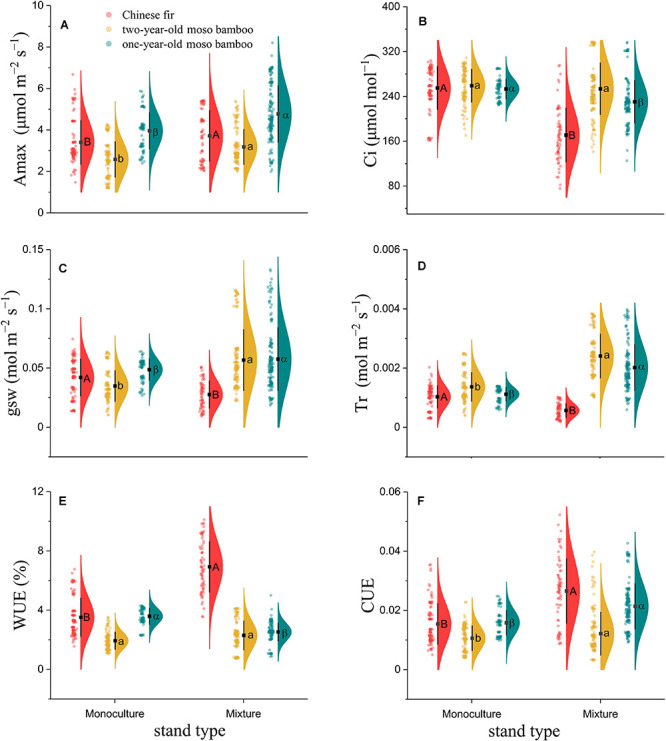
Raincloud plot of photosynthetic parameters among **(A)** Amax, **(B)** Ci, **(C)** gsw, **(D)** Tr, **(E)** WUE, and **(F)** CUE combined. The “cloud” represents the data distribution, and the “rain” represents the jittered raw data. The black point represents the mean distribution values, and the black line represents the standard deviations of the distributions. Capital letters indicate significant differences between Chinese fir under monoculture and mixed-stand conditions (*P* < 0.05). Lowercase letters indicate significant differences between 2-year-old moso bamboo under monoculture and mixed-stand conditions (*P* < 0.05). Greek letters indicate significant differences between 1-year-old moso bamboo under monoculture and mixed-stand conditions (*P* < 0.05).

The carbon assimilation of both Chinese fir and moso bamboo in MCM was significantly higher than that in PMB and PCF (Figure 2A). The photosynthetic parameters Amax, WUE, and CUE measured in Chinese fir trees grown in mixed stands (MCM) were 9.3, 82.6, and 70.7% higher, respectively, than those measured in Chinese fir monoculture plots (PCF) (*P* < 0.01; Figures 2A,E,F), whereas Ci, gsw, and Tr were 35.5, 34.4, and 44.7% lower, respectively, in MCM than in PCF (*P* < 0.001; Figures 2B,C,D). Compared with PMB, 1-year-old moso bamboo in MCM exhibited significantly higher Amax, gsw, Tr and CUE, by 20.5, 18.2, 88.4, and 35.2%, respectively (*P* < 0.001; Figures 2A,C,D,F). Similarly, compared with PMB, 2-year-old moso bamboo in MCM exhibited significantly higher Amax, gsw, Tr and CUE, by 23.3, 19.5, 61.6, and 15.5%, respectively (*P* < 0.001; Figures 2A,C,D,F). However, 1-year-old moso bamboo exhibited significantly lower Ci and WUE, by 9.1 and 29.8%, respectively, in MCM than in PMB (*P* < 0.001; Figures 2B,E). There was no significant differences in the Ci and WUE of 2-year-old moso bamboos grown in MCM and PMB (*P* > 0.05; Figures 2B,E).

### Chlorophyll a Fluorescence Parameters

**FIGURE 3:**
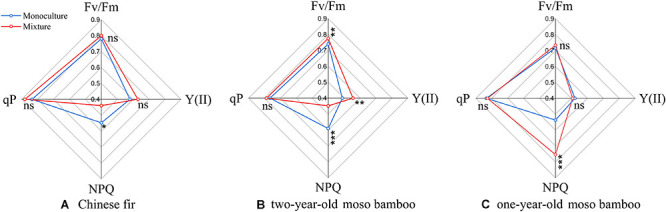
Radar plot showing various fluorescence parameters of the different stand types. **(A)** Chinese fir. **(B)** 2-year-old moso bamboo. **(C)** 1-year-old moso bamboo. Each line represents the average of 27 measurements per stand type. *P < 0.05, **P < 0.01, and ***P < 0.001.

The values of all fluorescence parameters (Fv/Fm, qP, and Y(II), with the exception of NPQ) of Chinese fir and two-year-old moso bamboo in MCM were slightly higher than those in PCF and PMB (*P* > 0.05; Figures 3A,B). NPQ was 19.8 and 24.1% lower, respectively, for Chinese fir and 2-year-old moso bamboo in MCM than in PCF and PMB (*P* < 0.05; Figures 3A,B), while NPQ was 40.4% higher in the 1-year-old moso bamboo in MCM than in PMB (*P* < 0.05; Figure 3C).

### Antioxidant Enzyme Activities

**FIGURE 4:**
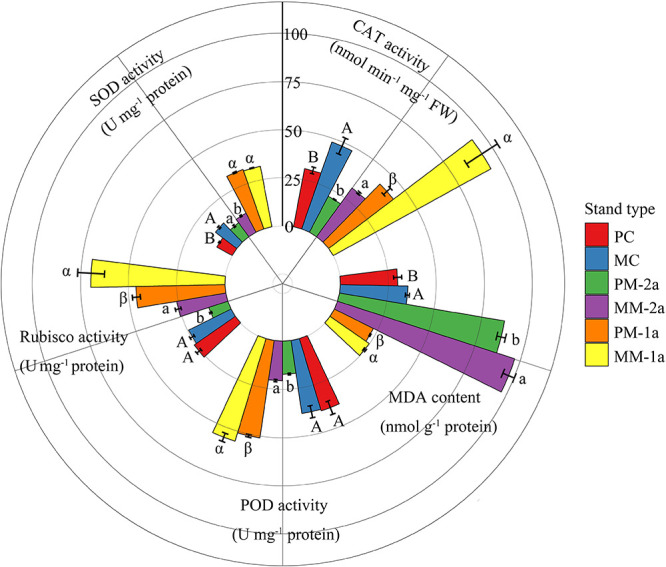
Antioxidant enzyme activities, Rubisco activity, and malondialdehyde (MDA) content in moso bamboo and Chinese fir leaves in different stand types. Capital letters indicate significant differences between Chinese fir under monoculture (PC) and mixed-stand (PM) conditions (*P* < 0.05). Lowercase letters indicate significant differences between 2-year-old moso bamboo under monoculture (PM-2a) and mixed-stand (MM-2a) conditions (*P* < 0.05). Greek letters indicate significant differences between 1-year-old (1a) moso bamboo under monoculture (PM-1a) and mixed-stand (MM-1a) conditions (*P* < 0.05). SOD: superoxide dismutase; POD: peroxidase; CAT: catalase.

Growing in MCM had significant effects on antioxidant enzyme activity and MDA content in Chinese fir and moso bamboo of both ages (Figure 4). Compared with PCF, MCM significantly increased SOD and CAT activity and MDA content in Chinese fir by 53.2, 55.4, and 17.6%, respectively (*P* < 0.001; Figure 4). Similarly, for 1-year-old moso bamboo, the POD, CAT and Rubisco activities and MDA content were 8.7, 121.4, 50.4, and 9.2% higher, respectively, in MCM than in PMB (*P* < 0.001; Figure 4). For 2-year-old moso bamboo, the SOD, POD, CAT and Rubisco activities and MDA content were 24.4, 19.9, 48.2, 149.8, and 11.4% higher, respectively, in MCM than in PMB (*P* < 0.001; Figure 4).

### Chlorophyll Content and Leaf Chemistry

**FIGURE 5:**
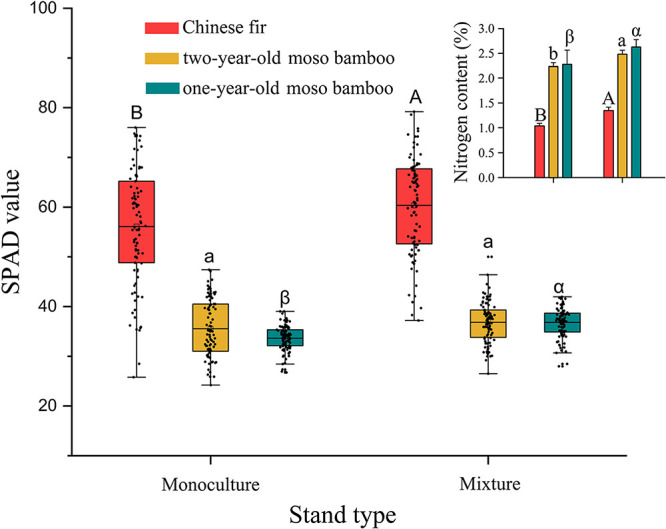
Chlorophyll content and leaf N content in moso bamboo and Chinese fir leaves in different stand types. Capital letters indicate significant differences between Chinese fir under monoculture and mixed-stand conditions (*P* < 0.05). Lowercase letters indicate significant differences between 2-year-old moso bamboo under monoculture and mixed-stand conditions (*P* < 0.05). Greek letters indicate significant differences between 1-year-old moso bamboo under monoculture and mixed-stand conditions (*P* < 0.05).

The chlorophyll contents of Chinese fir and 1-year-old moso bamboo in MCM were 7.5 and 3.7% higher, respectively, than those in PMB and PCF (*P* < 0.05; Figure 5). Similarly, the N content was 30.3, 15.3, and 4.6% higher among Chinese fir, 1-year-old and 2-year-old moso bamboo plants, respectively, in MCM than in PCF and PMB (*P* < 0.001; Figure 5).

## Discussion

### Effects of Mixed Growth on Photosynthesis

Photosynthesis is an indispensable component of plant activity, and it is the only process on earth that converts inorganic matter into organic matter and light energy into chemical energy (Wu et al., 2018b). The photosynthetic capability of plants reflects their productivity to a certain extent. We aimed to evaluate the effects of growing in a mixed stand on the physiological activity of Chinese fir and moso bamboo plantation trees under subtropical conditions. This information is relevant both for understanding how growing in mixed stands may affect the performance of moso bamboo and Chinese fir and for assessing the potential benefits of mixed stands in subtropical forests. Our results showed that growing in MCM significantly affected all photosynthetic parameters in both Chinese fir and moso bamboo, including Amax, Ci, gsw, Tr, WUE, and CUE. The increase in Amax supported our first hypothesis, according to which the plants growing in mixed stands would exhibit greater photosynthetic capacity than those growing in monocultures. Moreover, we observed that the reasons for the increase in Amax differed between moso bamboo and Chinese fir.

The increase in photosynthesis in Chinese fir in MCM was driven by its optimization of CO_2_ and water uptake when it was grown with moso bamboo. This explanation is supported by the observed increases in the WUE and CUE, which were higher in MCM than in PCF (Figures 2E,F and Supplementary Table 1). On the one hand, the increase in WUE and the reduction in transpiration may have also increased the competitive advantage of Chinese fir growing in environments where water is a limiting resource (Figures 2D,E; Andrés et al., 2017). On the other hand, a shadier environment proportioned by the taller moso bamboo may reduce incoming radiation in Chinese fir so that the damaging effects of excessive light energy on the photosynthetic complex was reduced. Meanwhile, transpiration rate was reduced, although lower stomatal conductance rates were keeping stable, thereby supporting higher WUE and photosynthesis (Liu et al., 2016). The higher CUE of the Chinese fir in MCM suggests that these trees maximized their use of carbon resources; this strategy may have increased their energy production and minimized the harmful effects of excessive light energy on the photosynthetic complex (He et al., 2011).

However, the Tr, gsw, and Ci were lower in Chinese fir growing in MCM than in Chinese fir growing in PCF. This result can be explained by the fact that Chinese fir exhibits strong physiological self-regulation abilities when adapting to water resource competition (Annicchiarico et al., 2015; Li et al., 2015). First, the decrease in the Tr and the increase in the WUE of Chinese fir in MCM may indicate that water stress in Chinese fir might occur due to interspecific competition under these conditions (Zhang et al., 2017a). Because of the shallow rooting depths (0–40 cm) of both moso bamboo (Chen et al., 2013; Shinohara et al., 2019) and Chinese fir (Fan, 2019; Liao et al., 2019), competition for soil water may induce water stress when the plants are taking up water from shallow soil layers (Schwendenmann et al., 2014). To tolerate water stress, complex physiological responses in Chinese fir that regulate photosynthetic productivity and promote survival are triggered (Xu, 2013). Stomatal closure is one of the first responses to water stress (Van Goethem et al., 2014; Nadal and Flexas, 2018); it allows plants to maintain an adequate water status by reducing transpiration to sustain critical physiological and biochemical processes (Han et al., 2019). In general, stomatal closure induced by water stress can reduce the photosynthetic capacity of plants (Xu, 2013), though our results indicated that moderate stomatal closure may benefit water preservation and promote WUE. To some extent, the high photosynthetic capacity of Chinese fir in the mixed stand (Amax = 3.7 μmol m^–2^ s^–1^ in the mixture; Figure 2A) may have contributed to its strong resistance to water stress (Annicchiarico et al., 2015; Li et al., 2015).

Second, a decrease in gsw usually leads to a decrease in the Ci and photosynthesis due to the limited CO_2_ diffusion from the atmosphere to the intercellular airspace. Fortunately, the decrease in photosynthesis caused by decreased gsw under drought conditions can be alleviated by maintaining the mesophyll conductance (gm) (Aranda et al., 2007; Han et al., 2016). Because the variability of gm has little or no effect on leaf water transpiration, it can affect CO_2_ diffusion from substomatal cavities to the carboxylation sites within chloroplasts (Flexas et al., 2012; Cano et al., 2014; Flexas, 2016; Han et al., 2016). It is possible that maintaining the gm can indirectly balance CO_2_ uptake and water loss, thereby achieving optimization between CO_2_ and water at the carboxylation sites in the chloroplast and contributing to improved photosynthesis and drought tolerance.

Since not all tree species utilize resources in the same manner, growth in mixed stands is more active than that in monocultures. The strategy of moso bamboo differs from those observed in Chinese firs for increasing its photosynthetic capacity. The Amax was much higher in both 1-year-old and 2-year-old moso bamboo growing in MCM than those growing in PMB. Because moso bamboo growing in MCM has a higher competitive advantage than Chinese fir for obtaining soil water from shallow soil layers due to its complex belowground rhizomatous clonal growth system (Song et al., 2017; Shinohara et al., 2019), moso bamboo in the MCM can more readily access soil water resources than that growing in the PMB, suggesting a release from intraspecific competition in the MCM. It is generally recognized that increasing soil water competition ability can help increase CO_2_ diffusion from the atmosphere to the carboxylation site due to stomatal opening and increased gm, which, in turn, contribute to an increase in Amax (Chaves and Oliveira, 2004; Ashraf and Harris, 2013). In addition, due to the tightly coupled relationship between leaf photosynthesis and Tr, the higher Tr of moso bamboo was expected to result in higher photosynthesis (Zhang et al., 2017a; Gu et al., 2019). The physiological integration of moso bamboo through the belowground rhizome system may promote its advantages in competing for soil moisture in mixed stands and thus higher productivity. Similarly, increased photosynthetic capacity and WUE were found in grass-legume mixtures (Liu et al., 2016) and in mixed conifer-hardwood forests (Fernández-de-Uña et al., 2016). Forrester et al. (2010, 2012) also found that the Amax and WUE of *Eucalyptus globulus* Labill increased in mixtures with *Acacia mearnsii* de Wildeman; several different processes can contribute to this response (Forrester et al., 2010; Forrester, 2015; Pretzsch et al., 2016). These findings are consistent with our results that the photosynthetic capacity and water stress responses of moso bamboo and Chinese fir in MCM were different from those in PMB and PCF. Hence, mixed stands may result in species interactions and increased interspecies competition. The fierce interspecies competition in mixed stands drives trees to achieve a competitive advantage in obtaining soil water resources through physiological self-regulation or interspecific morphological differences, which ultimately enhances their net photosynthetic capacity (Forrester, 2015; Forrester and Bauhus, 2016). Different strategies bring different benefits to plants as well as different risks. The risk of the photosynthesis improvement strategy in moso bamboo is that opening the stomata to increase Tr will be accompanied by a decrease in WUE (Figures 2C–E). As a result, moso bamboo may lose more water through transpiration, making it more difficult for Chinese fir to obtain soil water. For Chinese fir, the disadvantage of this lower stomatal conductance and lower transpiration water is its weakened capacity to compete for soil water resources under drought stress when growing in mixed stands with moso bamboo.

Previous studies have shown that mixed plantations have a higher carbon sequestration capacity and stand-level productivity than monocultures (Liang et al., 2016; Pretzsch et al., 2016; Huang et al., 2018) consistent with our results. However, mixed stands do not always promote the productivity of each component species in comparison to their performance in monocultures, perhaps due to changes in species proportions and inter-specific differences in biomass allocation patterns (Lee et al., 2003). In a pot trial with mixtures of Norway spruce (*P. abies* L.) and European beech, the former suffered a competitive disadvantage in mixtures, which had lower crown volumes, above-ground biomass and Amax compared to monocultures (Kozovits et al., 2005). Similarly, in another pot trial with mixtures of *Pinus taeda* and *Acer rubrum*, the *Acer rubrum* of biomass, height, Amax and biomass allocation to leaves were significantly lower in mixtures than in monocultures (Groninger et al., 1996). The reason for the difference photosynthesis capacity of the mixed plantation may be depended on the competitive ability of the admixed species and their influence on the availability and uptake of resources (Forrester et al., 2012). In addition, the stand density may be a critical factor affecting forest growth photosynthesis capacity (Forrester and Bauhus, 2016), and the changes in soil microorganism diversity and soil microbial activities in the mixed plantation may also contribute to the different photosynthetic capacity by improving soil nutrition conditions for plants (Wu et al., 2018a). In addition, these results may only represent the performance of plants in May. Long-term seasonal changes will be examined in the future study.

Our results showed that the interaction of mixture and species had significant effect on WUE and CUE, which may indicate that growing in mixed stands exacerbated the competitive for nutrition (such as water and nitrogen) between species. The previous study also confirmed that N addition alleviates the competitive effect between the introduced invasive plant *Robinia pseudoacacia* L. and the native tree *Quercus acutissima* Carr. (Luo et al., 2014).

### Effects of Mixed Growth on Fluorescence Parameters

Chlorophyll fluorescence, as an important component of plant photosynthesis, is widely used to study photosynthetic performance in leaves, and it can provide useful information about physical changes in pigment-protein complexes and the electron transport rate through PSII (Baker and Rosenqvist, 2004). Chlorophyll fluorescence parameters [Fv/Fm, Y(II), NPQ, and qN] are sensitive to water stress (Pan et al., 2012; Van Goethem et al., 2013; Yuan et al., 2016; Wu et al., 2018b) and photosynthetic status (Man et al., 2017; Rudzani et al., 2017). Thus, we further investigated whether the photochemical response can detect the potential stresses and changes in photosynthetic status induced in mixed stands.

In our study, all fluorescence parameters of moso bamboo and Chinese fir were slightly higher in MCM than in PCF and PMB, and only the NPQ of Chinese fir and 2-year-old moso bamboo was significantly lower in MCM. Among the fluorescence parameters, the value of Fv/Fm can be used to detect damage to PSII and to the photosynthetic efficiency of leaves resulting from environmental stress (Yuan et al., 2016; Wu et al., 2018b). Numerous studies have confirmed that when the Fv/Fm decreases due to water stress, it may destroy the PSII oxygen-evolving complex and the PSII reaction centers and in turn degrade the D1 protein (Van Goethem et al., 2013; Man et al., 2017; Rudzani et al., 2017). Our photosynthetic data may indicate that Chinese fir suffered from water stress in MCM, but this was not reflected in its Fv/Fm, which was slightly higher in MCM than PCF. The reason for this discrepancy may be that a decrease in Fv/Fm can be detected only when structural damage occurs in the photosystem II primary photochemistry, resulting in a decrease in the photosynthetic rate (Wu et al., 2018b).

The parameter qP represents the photochemical capacity and the number of QA oxidation states in PSII under light adaptation. A higher qP value is advantageous for the separation of electric charges in the reaction center and is beneficial to electron transport and PSII yield (Rudzani et al., 2017). The slight increases in Fv/Fm, qP, and Y(II) in both Chinese fir and moso bamboos of both ages in MCM were consistent with the increased photosynthetic rate, which may indicate that mixed plantations can protect plant photochemical efficiency from the damage caused by environmental stresses, such as water stress (Wu et al., 2018b). The reason for the slightly higher fluorescence parameters of each species may attribute to higher leaf nitrogen concentrations in moso bamboo and Chinese fir growing in the mixed stand (Figure 5) which is mainly involved in light energy capture, electron transfer and carboxylation processes in photosynthesis (Forrester et al., 2012).

The lower NPQ in Chinese fir and 2-year-old moso bamboo in MCM indicates that the light energy captured by leaves was fully used for photosynthesis (Liu et al., 2016), increasing photoprotection for the plants and reducing the risk of light damage (Zhang et al., 2017a; Demmig-Adams et al., 1996). However, the significantly higher NPQ of the 1-year-old moso bamboo in the MCM suggested that thermal energy dissipation was activated to dissipate the excess light energy, thereby preventing ROS generation and maintaining the balance between the absorption and consumption of light energy under a moderate water deficit (Yi et al., 2018). The above results suggested that MCM improved the light use efficiency of each component compared to that of PCF and PMB. In addition, these results also suggest that Chinese fir and 2-year-old moso bamboo have a higher light use efficiency compared to 1-year-old moso bamboo. The different responses of Chinese fir and 2-year-old moso bamboo and 1-year-old moso bamboo can be explained by the differences in growth stage and environmental adaptation mechanism (Wen et al., 2011).

While Fv/Fm has served as an indicator of water stress in previous studies (Van Goethem et al., 2013; Yuan et al., 2016; Zhang et al., 2017b; Wu et al., 2018b), our study also further supported previous results showing that NPQ is very sensitive to light use efficiency and could be a useful indicator of the impacts of mixed plantations (Mathobo et al., 2017; Zhang et al., 2017a).

### Effects of Mixed Growth on Physiological and Biochemical Resistance

ROS are often produced in chloroplasts during photosynthesis. Under normal circumstances, a balance exists between ROS production and elimination in plants (Wu et al., 2020). However, water stress can change the electron transport chain and exacerbate the production of ROS such as O_2_ and H_2_O_2_ (Man et al., 2017), which ultimately results in the disruption of this balance. This effect is harmful to organelles, including chloroplasts, mitochondria and peroxisomes, and may damage the photosynthetic apparatus through the oxidation of lipids, proteins, carbohydrates and nucleic acids (Mittler, 2002; Wu et al., 2018b). To minimize the occurrence of oxidative damage, plants have developed an array of protective and repair systems; these systems include the antioxidative system, which involves enzymes such as SOD, POD, and CAT that react with ROS and keep ROS levels low (Imlay, 2003; Reddy et al., 2004).

Cell membrane lipid peroxidation is an important index of plant damage caused by water deficit. It occurs due to the accumulation of ROS when the balance between ROS generation and removal is disturbed. Thus, the content of MDA (a product of lipid peroxidation) is often used to assess the extent of oxidative damage in plant tissue (Noctor and Foyer, 1998; Mittler, 2002). An increase in MDA content suggests that lipid peroxidation caused by ROS accumulation has resulted in cellular damage. Our results showed an increase in the MDA concentration of both species in MCM, which may indicate that oxidative stress caused cellular damage and therefore metabolic disorders in these plants (Wu et al., 2020). However, an effective antioxidative system in plants can minimize oxidative stress levels and protect their tissues. The activity of SOD catalyses the disproportionation of O_2_^–^ to molecular oxygen and H_2_O_2_, which is further reduced to water by CAT; these processes maintain a very low steady-state concentration of O_2_^–^ and H_2_O_2_ and minimize the production of hydroxyl radicals (Yi et al., 2018). Similarly, POD activity plays an important role in antioxidative protection. We observed that the activities of SOD, CAT, and POD were higher in MCM than in PCF and PMB, suggesting that antioxidative enzymes were mobilized to protect the plant tissue from oxidative damage, which showed that mixed plantations may induce water stress conditions (Wu et al., 2020). In addition, future studies should focus on sap flux density to quantify the water stress of both species in the different mixture conditions. Our results indicate that growing in mixed plantations can hone the ability of plant antioxidant systems, stimulate cells to enter a sensitized state, induce plants to effectively control excessive ROS concentrations and increase the ability of plants to resist stress.

Rubisco activity has been considered a useful indicator for breeding programmes aiming to enhance plant WUE as well as yield (Hirel et al., 2007). In our study, an increase in Rubisco activity in MCM was considered beneficial for plant survival under water stress conditions because a positive relationship between leaf Rubisco content and Amax has been reported in most C_3_ plants (Taub, 2010; Makino, 2011). An increase in the rate of the Rubisco-catalyzed reaction has been reported to occur due to changes in CO_2_ availability at the chloroplast level or in the availability of the Rubisco substrate or due to the activation of Rubisco (Han et al., 2019).

### Effects of Mixed Growth on Leaf N and Chlorophyll Contents

The results also showed that Chinese fir and moso bamboo of both ages had significantly higher chlorophyll and leaf nitrogen contents in MCM than PMB and PCF, which corresponds to the high photosynthesis rate in MCM (Liu et al., 2016). Because the majority of leaf nitrogen is used in the photosynthetic apparatus, a high leaf nitrogen content results in an increase in the number of thylakoids and an increase in thylakoid proteins in chloroplasts (Evans, 1989). A strong, positive, causal correlation between the photosynthetic rate of the leaf and the leaf nitrogen content has been reported (Xu, 2013). In other words, the increase in leaf nitrogen content in mixed plantations can increase the maximum photosynthetic rates of the trees. In addition, the chlorophyll content per unit of leaf area, the specific activity of RuBP carboxylase and the electron transport capacity increase with increasing nitrogen content, which improves the plant photosynthetic capacity (Sugiharto et al., 1990; Nakaji et al., 2001; Xu, 2013). Küppers (1996) found that *E. delegatensis* R. T. Bak. saplings growing with *Acacia dealbata* Link. had higher leaf N levels in a native forest. Similarly, Forrester et al. (2012) observed that the leaf nitrogen content of *Eucalyptus globulus* was greater in mixed plantations than in monocultures. These results are consistent with our results, in which the leaf nitrogen content of Chinese fir and both ages of moso bamboo in MCM was significantly higher than that in PMB and PCF. The researchers found that the possible cause of this increase was that higher N availability in the mixed stands resulted from increased rates of N_2_-fixation and accelerated rates of nutrient cycling (Forrester et al., 2005; Forrester et al., 2012). However, Amax did not increase with increasing leaf nitrogen in *Acacia mearnsii* in a mixed stand as it did in a monoculture (Forrester et al., 2012), and others found no relationships, suggesting that leaf nitrogen content was in excess of photosynthetic requirements and that Amax was limited by other factors (Warren et al., 2000; Close et al., 2004). The difference of the facilitative influence of mixed planting was caused by species specific (Küppers, 1996; Forrester et al., 2012).

## Conclusion

The increase in the net photosynthetic capacity of the moso bamboo and Chinese fir grown in mixed stands can primarily be attributed to their (i) more competitive strategies for soil water use, (ii) stronger antioxidant activity, and (iii) higher leaf total nitrogen and chlorophyll contents. The results of our study indicate that mixed stands can significantly alter the photosynthetic capacity and physiological stress resistance of plants. These findings may provide a potential approach to increasing the carbon sequestration capacity to mitigation of global warming effect and serve as reasonable guidance for the cultivation of Chinese fir and moso bamboo plantations.

## Data Availability Statement

The original contributions presented in the study are included in the article/Supplementary Material, further inquiries can be directed to the corresponding author/s.

## Author Contributions

CP analyzed the data, drafted the manuscript, and participated in collecting the experiment data. GZ and YJS designed this experiment. YDS and ZW conducted the field and laboratory experiments. TM and CL contributed to revision of the manuscript. All authors discussed the results and revised the manuscript. All authors contributed to the article and approved the submitted version.

## Conflict of Interest

The authors declare that the research was conducted in the absence of any commercial or financial relationships that could be construed as a potential conflict of interest.

## References

[B1] Andrés E. G. D. Camarero J. J. Blanco J. A. Imbert J. B. Lo Y. Sangüesa-Barreda G. (2017). Tree-to-tree competition in mixed European beech-Scots pine forests has different impacts on growth and water-use efficiency depending on site conditions. *J. Ecol.* 106 59–75. 10.1111/1365-2745.12813

[B2] Annicchiarico P. Barrett B. Brummer E. C. Julier B. Marshall A. H. (2015). Achievements and challenges in improving temperate perennial forage legumes. *Crit. Rev. Plant Sci.* 34 327–380. 10.1080/07352689.2014.898462

[B3] Aranda I. Pardos M. Puértolas J. Jiménez M. D. Pardos J. A. (2007). Water-use efficiency in cork oak (Quercus suber) is modified by the interaction of water and light availabilities. *Tree Physiol.* 27 671–677. 10.1093/treephys/27.5.671 17267358

[B4] Ashraf M. Harris P. J. C. (2013). Photosynthesis under stressful environments: an over-view. *Photosynthetica* 51 163–190. 10.1007/s11099-013-0021-6

[B5] Bai S. Conant R. Zhou G. Wang Y. X. Wang N. Li Y. (2016). Effects of Moso bamboo encroachment into native, broad-leaved forests on soil carbon and nitrogen pools. *Sci. Rep.* 6:31480. 10.1038/srep31480 27526781 PMC4985758

[B6] Baker N. R. Rosenqvist E. (2004). Applications of chlorophyll fluorescence can improve crop production strategies: an examination of future possibilities. *J. Exp. Bot.* 55 1607–1621. 10.1093/jxb/erh196 15258166

[B7] Cano F. J. Lopez R. Warren C. R. (2014). Implications of the mesophyll conductance to CO2 for photosynthesis and water-use efficiency during long-term water stress and recovery in two contrasting Eucalyptus species. *Plant Cell Environ.* 37 2470–2490. 10.1111/pce.12325 24635724

[B8] Chance B. Maehly A. C. (1955). Assay of catalases and peroxidases. *Methods. Enzymol.* 2 764–775. 10.1016/S0076-6879(55)02300-8 13193536

[B9] Chaves M. Oliveira M. (2004). Mechanisms underlying plant resilience to water deficits: prospects for water saving agriculture. *J. Exp. Bot.* 55 2365–2384. 10.1093/jxb/erh269 15475377

[B10] Chen H. Feng Y. Zhou J. Xu Z. Lian C. Guo Q. (2013). Root biomass distribution and seasonal variation of Phyllostachys edulis. *Ecol. Environ. Sci.* 22 1678–1681.

[B11] Chen L. C. Wang S. L. Wang P. Kong C. H. (2014). Autoinhibition and soil allelochemical (cyclic dipeptide) levels in replanted Chinese fir (Cunninghamia lanceolata) plantations. *Plant Soil.* 374 793–801. 10.1007/s11104-013-1914-7

[B12] Close D. C. Battaglia M. Davidson N. J. Beadle C. L. (2004). Within-canopy gradients of nitrogen and photosynthetic activity of eucalyptus nitens and eucalyptus globulus in response to nitrogen nutrition. *Aust. J. Bot.* 52 133–140. 10.1071/bt03027

[B13] Demmig-Adams B. Adams W. W. Barker D. H. Logan B. A. Bowling D. R. Verhoeven A. S. (1996). Using chlorophyll fluorescence to assess the fraction of absorbed light allocated to thermal dissipation of excess excitation. *Physiol. Plant.* 98 253–264. 10.1034/j.1399-3054.1996.980206.x 11841302

[B14] Evans J. (1989). Photosynthesis and nitrogen relationships in leaves of C*3* plants. *Oecologia* 78 9–19. 28311896 10.1007/BF00377192

[B15] Fan S. R. (2019). Study on the root spatial distribution pattern of 5 years old Cunninghamia lanceolate and Liquidambar mixed forest. *For. Prospect Design* 2 39–45.

[B16] Fernández-de-Uña L. McDowell N. Cañellas I. Gea-Izquierdo G. (2016). Disentangling the effect of competition, CO2 and climate on intrinsic water-use efficiency and tree growth. *J. Ecol.* 104 678–690. 10.1111/1365-2745.12544

[B17] Flexas J. (2016). Genetic improvement of leaf photosynthesis and intrinsic water use efficiency in C3 plants: why so much little success? *Plant Sci.* 251 155–161. 10.1016/j.plantsci.2016.05.002 27593473

[B18] Flexas J. Barbour M. M. Brendel O. Cabrera H. M. Carriquí M. Díaz-Espejo A. (2012). Mesophyl diffusion conductance to CO2: an unappreciated central player in photosynthesis. *Plant Sci.* 193-194 70–84. 10.1016/j.plantsci.2012.05.009 22794920

[B19] Forrester D. I. (2015). Transpiration and water-use efficiency in mixed-species forests versus monocultures: effects of tree size, stand density and season. *Tree Physiol.* 35 289–304. 10.1093/treephys/tpv011 25732385

[B20] Forrester D. I. Albrecht A. T. (2014). Light absorption and light-use efficiency in mixtures of abies alba and picea abies along a productivity gradient. *For. Ecol. Manage.* 328 94–102. 10.1016/j.foreco.2014.05.026

[B21] Forrester D. I. Bauhus J. (2016). A review of processes behind diversity—productivity relationships in forests. *Curr. For. Rep.* 2 45–61. 10.1007/s40725-016-0031-2

[B22] Forrester D. I. Bauhus J. Cowie A. L. (2005). Nutrient cycling in a mixed-species plantation of Eucalyptus globulus and Acacia mearnsii. *Can. J. For. Res.* 35 2942–2950.

[B23] Forrester D. I. Lancaster K. Collopy J. J. Warren C. R. Tausz M. (2012). Photosynthetic capacity of Eucalyptus globulus is higher when grown in mixture with Acacia mearnsii. *Trees* 26 1203–1213. 10.1007/s00468-012-0696-5

[B24] Forrester D. I. Theiveyanathan S. Collopy J. J. Marcar N. E. (2010). Enhanced water use efficiency in a mixed Eucalyptus globules and Acacia mearnsii plantation. *For. Ecol. Manage.* 259 1761–1770. 10.1016/j.foreco.2009.07.036

[B25] Genty B. Briantais J. M. Baker N. R. (1989). The relationship between the quantum yield of photosynthetic electron transport and quenching of chlorophyll fluorescence. *Biochim. Biophys. Acta* 990 87–92. 10.1016/S0304-4165(89)80016-9

[B26] Giannopolitis C. N. Ries S. K. (1977). Superoxide dismutases: I. Occurrence in higher plants. *Plant Physiol.* 59 309–314. 10.1104/pp.59.2.309 16659839 PMC542387

[B27] Groninger J. W. Seiler J. R. Zedaker S. M. Berrang P. C. (1996). Effects of CO2 concentration and water availability on growth and gas exchange in greenhouse-grown miniature stands of Loblolly Pine and Red Maple. *Funct. Ecol.* 10 708–716. 10.2307/2390505

[B28] Gu D. He W. Huang K. Otieno D. Zhou C. M. He C. X. (2019). Transpiration of Moso bamboo in southern China is influenced by ramet age, phenology, and drought. *For. Ecol. Manage.* 450:117526. 10.1016/j.foreco.2019.117526

[B29] Guan F. Tang X. Fan S. Zhao J. Peng C. (2015). Changes in soil carbon and nitrogen stocks followed the conversion from secondary forest to Chinese fir and Moso bamboo plantations. *Catena* 133 455–460. 10.1016/j.catena.2015.03.002

[B30] Han J. Lei Z. Zhang Y. Yi X. Zhang W. Zhang Y. (2019). Drought-introduced variability of mesophyll conductance in Gossypium and its relationship with leaf anatomy. *Physiol. Plant* 166 873–887. 10.1111/ppl.12845 30264467

[B31] Han J. M. Meng H. F. Wang S. Y. Jiang C. D. Liu F. Zhang W. F. (2016). Variability of mesophyll conductance and its relationship with water use efficiency in cotton leaves under drought pretreatment. *J. Plant Physiol.* 194 61–71. 10.1016/j.jplph.2016.03.014 27101723

[B32] He G. Zhang J. Hu X. Wu J. (2011). Effect of aluminum toxicity and phosphorus deficiency on the growth and photosynthesis of oil tea (Camellia oleifera Abel.) seedlings in acidic red soils. *Acta Physiol. Plant* 33 1285–1292. 10.1007/s11738-010-0659-7

[B33] Hirel B. Le Gouis J. Ney B. Gallais A. (2007). The challenge of improving nitrogen use efficiency in crop plants: towards a more central role for genetic variability and quantitative genetics within integrated approaches. *J. Exp. Bot.* 58 2369–2387. 10.1093/jxb/erm097 17556767

[B34] Huang Y. Chen Y. Castro-Izaguirre N. Baruffol M. Brezzi M. Lang A. (2018). Impacts of species richness on productivity in a large-scale subtropical forest experiment. *Science* 362 80–83. 10.1126/science.aat6405 30287660

[B35] Imlay J. A. (2003). Pathways of oxidative damage. *Annu. Rev. Microbiol.* 57 395–418. 10.1146/annurev.micro.57.030502.090938 14527285

[B36] Kelty M. J. (2006). The role of species mixtures in plantation forestry. *For. Ecol. Manage.* 233 195–204. 10.1016/j.foreco.2006.05.011

[B37] Kozovits A. R. Matyssek R. Blaschke H. Göttlein A. Grams T. E. E. (2005). Competition increasingly dominates the responsiveness of juvenile beech and spruce to elevated CO2 and/or O3 concentrations throughout two subsequent growing seasons. *Glob. Change Biol.* 11 1387–1401. 10.1111/j.1365-2486.2005.00993.x

[B38] Küppers B. I. L. (1996). Nitrogen and Rubisco contents in eucalypt canopies as affected by Acacia neighbourhood. *Plant Physiol. Biochem.* 34 753–760.

[B39] Lee T. D. Reich P. B. Tjoelker M. G. (2003). Legume presence increases photosynthesis and N concentrations of co-occurring non-fixers but does not moderate their responsiveness to carbon dioxide enrichment. *Oecologia* 137 22–31. 10.1007/s00442-003-1309-1 12802677

[B40] Li C. Shi Y. J. Zhou G. M. Zhou Y. F. Xu L. Tong L. (2018). Effects of different management approaches on soil carbon dynamics in Moso bamboo forest ecosystems. *Catena* 169 59–68. 10.1016/j.catena.2018.05.031

[B41] Li C. Wang K. (2003). Differences in drought responses of three contrasting *Eucalyptus microtheca* F. Muell. Populations. *For. Ecol. Manage.* 179 377–385. 10.1016/S0378-1127(02)00552-2

[B42] Li Q. Song Y. Li G. Yu P. Wang P. Zhou D. (2015). Grass-legume mixtures impact soil N, species recruitment, and productivity in temperate steppe grassland. *Plant Soil* 394 271–285. 10.1007/s11104-015-2525-2

[B43] Li Y. Zhang J. Chang S. Jiang P. Zhou G. Fu S. (2013). Long-term intensive management effects on soil organic carbon pools and chemical composition in Moso bamboo (*Phyllostachys pubescens*) forests in subtropical China. *For. Ecol. Manage.* 303 121–130. 10.1016/j.foreco.2013.04.021

[B44] Liang J. Crowther T. W. Picard N. Wiser S. Zhou M. Alberti G. (2016). Positive biodiversity-productivity relationship predominant in global forests. *Science* 354:aaf8957. 10.1126/science.aaf8957 27738143

[B45] Liao Y. Fan H. Wei X. Wu J. Duan H. Fu X. (2019). Competition increased fine root biomass in Chinese fir (Cunninghamia lanceolata) plantations in Subtropical China. *For. Ecol. Manage.* 435 151–157. 10.1016/j.foreco.2018.12.035

[B46] Liu B. Liu Q. Daryanto S. Guo S. Huang Z. Wang Z. (2018). Responses of Chinese fir and Schima superba seedlings to light gradients: Implications for the restoration of mixed broadleaf-conifer forests from Chinese fir monocultures. *For. Ecol. Manage.* 41 51–57. 10.1016/j.foreco.2018.03.033

[B47] Liu M. Gong J. Pan Y. Luo Q. Zhai Z. Xu S. (2016). Effects of grass-legume mixtures on the production and photosynthetic capacity of constructed grasslands in Inner Mongolia. *China. Crop Pasture Sci.* 67 1188–1198. 10.1071/CP16063

[B48] Luo Y. Guo W. Yuan Y. Liu J. Du N. Wang R. (2014). Increased nitrogen deposition alleviated the competitive effects of the introduced invasive plant *Robinia pseudoacacia* on the native tree Quercus acutissima. *Plant Soil* 385 63–75. 10.1007/s11104-014-2227-1

[B49] Makino A. (2011). Photosynthesis, grain yield, and nitrogen utilization in rice and wheat. *Plant Physiol.* 155 125–129. 10.1104/pp.110.165076 20959423 PMC3014227

[B50] Man J. Yu Z. Shi Y. (2017). Radiation interception, chlorophyll fluorescence and senescence of flag leaves in winter wheat under supplemental irrigation. *Sci. Rep.* 7:7767. 10.1038/s41598-017-07414-2 28798391 PMC5552736

[B51] Mathobo R. Marais D. Steyna J. M. (2017). The effect of drought stress on yield, leaf gaseous exchange and chlorophyll fluorescence of dry beans (*Phaseolus vulgaris* L.). *Agric Water Manage.* 180 118–125. 10.1016/j.agwat.2016.11.005

[B52] Mittler R. (2002). Oxidative stress, antioxidants and stress tolerance. *Trends Plant Sci.* 7 405–410. 10.1016/S1360-1385(02)02312-9 12234732

[B53] Murchie E. H. Lawson T. (2013). Chlorophyll fluorescence analysis: a guide to good practice and understanding some new applications. *J. Exp. Bot.* 64 3983–3998. 10.1093/jxb/ert208 23913954

[B54] Nadal M. Flexas J. (2018). “Mesophyll conductance to CO2 diffusion: effects of drought and opportunities for improvement,” in *Water Scarcity and Sustainable Agriculture in Semiarid Environment*, eds García-T ejero I. F. Durán-Zuazo V. H. (London: Elsevier, Academic Press), 403–438.

[B55] Nakaji T. Fukami M. Dokiya Y. Izuta T. (2001). Effects of high nitrogen load on growth, photosynthesis and nutrient status of Cryptomeria japonica and Pinus densiflora seedlings. *Trees* 15 453–461. 10.1007/s00468-001-0130-x

[B56] Noctor G. Foyer C. H. (1998). Ascorbate and glutathione: keeping active oxygen under control. *Annu. Rev. Plant Physiol. Plant Mol. Biol.* 49 249–279. 10.1146/annurev.arplant.49.1.249 15012235

[B57] Pan J. Lin S. Woodbury N. W. (2012). Bacteriochlorophyll excited-state quenching pathways in bacterial reaction centers with the primary donor oxidized. *J. Phys. Chem. B* 116 2014–2022. 10.1021/jp212441b 22229638

[B58] Payton P. Webb R. Kornyeyev D. Allen R. Holaday A. S. (2001). Protecting cotton photosynthesis during moderate chilling at high light intensity by increasing chloroplastic antioxidant enzyme activity. *J. Exp. Bot.* 52 2345–2354. 10.1093/jexbot/52.365.2345 11709584

[B59] Pretzsch H. Del Río M. Schütze G. Ammer C. H. Annighöfer P. Avdagic A. (2016). Mixing of Scots pine (*Pinus sylvestris* L.) and European beech (*Fagus sylvatica* L.) enhances structural heterogeneity, and the effect increases with water availability. *For. Ecol. Manag.* 373 149–166. 10.1016/j.foreco.2016.04.043

[B60] Reddy A. R. Chaitanya K. V. Vivekanandan M. (2004). Drought-induced responses of photosynthesis and antioxidant metabolism in higher plants. *J. Plant Physiol.* 161 1189–1202. 10.1016/j.jplph.2004.01.013 15602811

[B61] Rudzani M. Diana M. Joachim M. S. (2017). The effect of drought stress on yield, leaf gaseous exchange and chlorophyll fluorescence of dry beans (Phaseolus vulgaris L.). *Agric. Water Manage.* 1 118–125. 10.1016/j.agwat.2016.11.005

[B62] Schwendenmann L. Pendall E. Sanchez-Bragado R. Kunert N. Hölscher D. (2014). Tree water uptake in a tropical plantation varying in tree diversity: interspecific differences, seasonal shifts and complementarity. *Ecohydrology* 8 1–12. 10.1002/eco.1479

[B63] SFAPRC (2015). *Forest Resources in China-The 8th National Forest Inventory.* Beijing: States Forestry Administration, PR China.

[B64] Shinohara Y. Misumi Y. Kubota T. Nankod K. (2019). Characteristics of soil erosion in a moso-bamboo forest of western Japan: Comparison with a broadleaved forest and a coniferous forest. *Catena* 172 451–460. 10.1016/j.catena.2018.09.011

[B65] Song Q. N. Lu H. Liu J. Yang J. Yang G. Y. Yang Q. P. (2017). Accessing the impacts of bamboo expansion on NPP and N cycling in evergreen broadleaved forest in subtropical China. *Sci. Rep.* 7:40383. 10.1038/srep40383 28067336 PMC5220298

[B66] Song X. Jiang H. Zhang Z. Zhou G. Zhang S. Peng C. (2014). Interactive effects of elevated UV-B radiation and N deposition on decomposition of Moso bamboo litter. *Soil Biol. Biochem.* 69 11–16. 10.1016/j.soilbio.2013.10.036

[B67] Song X. Peng C. Zhou G. Gu H. Li Q. Zhang C. (2016). Dynamic allocation and transfer of non-structural carbohydrates, a possible mechanism for the explosive growth of Moso bamboo (Phyllostachys heterocycla). *Sci. Rep.* 6:25908. 10.1038/srep25908 27181522 PMC4867622

[B68] Song X. Zhou G. Gu H. Li Q. (2015). Management practices amplify the effects of N deposition on leaf litter decomposition of the Moso bamboo forest. *Plant Soil* 395 391–400. 10.1007/s11104-015-2578-2

[B69] Sugiharto B. Miyata K. Nakamoto H. Sasakawa H. Sugiyama T. (1990). Regulation of expression of carbon-assimilating enzymes by nitrogen in maize leaf. *Plant Physiol.* 92 963–969. 16667412 10.1104/pp.92.4.963 PMC1062402

[B70] Tang X. Xia M. Pérez-Cruzado C. Guan F. Fan S. (2017). Spatial distribution of soil organic carbon stock in Moso bamboo forests in subtropical China. *Sci. Rep.* 7:42640. 10.1038/srep42640 28195207 PMC5307386

[B71] Taub D. (2010). Effects of rising atmospheric concentrations of carbon dioxide on plants. *Nat. Educ. Knowl.* 3:21.

[B72] Tu L. H. Peng Y. Chen G. Hu H. Xiao Y. Hu T. (2014). Direct and indirect effects of nitrogen additions on fine root decomposition in a subtropical bamboo forest. *Plant Soil* 389 273–288. 10.1007/s11104-014-2353-9

[B73] Ueda K. (1960). Studies on the physiology of bamboo, with reference to practical application. *Bull Kyoto Univ. For.* 30:169.

[B74] Van Goethem D. De Smedt S. Valcke R. Potters G. Samson R. (2013). Seasonal, diurnal, and vertical variation of chlorophyll fluorescence on Phyllostachys humilis in Ireland. *PLoS One* 8:e0072145. 10.1371/journal.pone.0072145 23967282 PMC3744462

[B75] Van Goethem D. Potters G. De Smedt S. Gu L. Samson R. (2014). Seasonal, diurnal, and vertical variation in photosynthetic parameters in Phyllostachys humilis bamboo plants. *Photosynth. Res.* 120 331–346. 10.1007/s11120-014-9992-9 24585025

[B76] Wang Q. Wang S. Zhang J. (2009). Assessing the effects of vegetation types on carbon storage fifteen years after reforestation on a Chinese fir site. *For. Ecol. Manage.* 25 1437–1441. 10.1016/j.foreco.2009.06.050

[B77] Wang X. M. Wang X. K. Su Y. B. Zhang H. X. (2019). Land pavement depresses photosynthesis in urban trees especially under drought stress. *Sci. Total Environ.* 653 120–130. 10.1016/j.scitotenv.2018.10.281 30408660

[B78] Wang Y. X. Bai S. B. Binkley D. Zhou G. M. Fang F. Y. (2016). The independence of clonal shoot’s growth from light availability supports moso bamboo invasion of closed-canopy forest. *For. Ecol. Manage.* 368 105–110. 10.1016/j.foreco.2016.02.037

[B79] Wang Y. X. Zhu X. D. Bai S. B. Zhu T. T. Qiu W. T. You Y. J. (2018). Effects of forest regeneration practices on the flux of soil CO2 after clear-cutting in subtropical China. *J. Environ. Manag.* 212 332–339. 10.1016/j.jenvman.2018.02.038 29453118

[B80] Warren C. R. Adams M. A. Chen Z. (2000). Is photosynthesis related to concentrations of nitrogen and Rubisco in leaves of Australian native plants? *Aust. J. Plant Physiol.* 27 407–416. 10.1071/PP98162

[B81] Wen G. Zhang L. Zhang R. Cao Z. Zhou G. Huang H. (2011). Temporal and spatial dynamics of carbon fixation by Mosso bamboo (Phyllostachys pubescens) in subtropical China. *Bot. Rev.* 77 271–277. 10.1007/s12229-011-9068-x 21957317 PMC3169764

[B82] Wu C. Mo Q. Wang H. Zhang Z. Huang G. Ye Q. (2018a). Moso bamboo (phyllostachys edulis (carriere) j. houzeau) invasion affects soil phosphorus dynamics in adjacent coniferous forests in subtropical china. *Ann. For. Sci.* 75 1–11. 10.1007/s13595-018-0703-0

[B83] Wu J. Q. Wang Y. X. Yang Y. Zhu T. T. Zhu X. D. (2014). Effects of crop tree release on stand growth and stand structure of Cunninghamia lanceolata plantation. *Chin. J. Appl. Ecol.* 26 340–348. 26094445

[B84] Wu L. Deng Z. Cao L. Meng L. (2020). Effect of plant density on yield and Quality of perilla sprouts. *Sci. Rep.* 10:9937. 10.1038/s41598-020-67106-2 32555363 PMC7303116

[B85] Wu Z. Z. Ying Y. Q. Zhang Y. B. Bi Y. F. Wang A. K. Du X. H. (2018b). Alleviation of drought stress in Phyllostachys edulis by N and P application. *Sci. Rep.* 8:228. 10.1038/s41598-017-18609-y 29321617 PMC5762838

[B86] Xu D. Q. (2013). *The Science of Photosynthesis.* Beijing: Science Press.

[B87] Yang Y. Wang L. Yang Z. Xu C. Xie J. Chen G. (2018). Large ecosystem service benefits of assisted natural regeneration. *J. Geophys. Res. Biogeosci.* 123 676–687. 10.1002/2017JG004267

[B88] Yi X. P. Zhang Y. L. Yao H. S. Han J. M. Chow W. S. Fan D. Y. (2018). Changes in activities of both photosystems and the regulatory effect of cyclic electron flow in field-grown cotton (*Gossypium hirsutum* L) under water deficit. *J. Plant Physiol.* 220 74–82. 10.1016/j.jplph.2017.10.011 29156245

[B89] Yuan S. Zhang Z. Zheng C. Zhao Z. Wang Y. Feng L. (2016). *Arabidopsis* cryptochrome 1 functions in nitrogen regulation of flowering. *Proc. Nat. Acad. Sci. U.S.A.* 113 7661–7666. 10.1073/pnas.1602004113 27325772 PMC4941442

[B90] Zhang H. Zhou G. Wang Y. Bai S. Sun Z. Berninger F. (2019). Thinning and species mixing in Chinese fir monocultures improve carbon sequestration in subtropical China. *Eur. J. For. Res.* 138 433–443. 10.1007/s10342-019-01181-7

[B91] Zhang H. Zhuang S. Qian H. Wang F. Ji H. (2015). Spatial variability of the topsoil organic carbon in the Moso bamboo forests of southern China in association with soil properties. *PLoS One* 10:e0119175. 10.1371/journal.pone.0119175 25789615 PMC4366393

[B92] Zhang R. Wu J. Li Q. Hänninen H. Peng C. Yao H. (2017a). Nitrogen deposition enhances photosynthesis in Moso bamboo but increases susceptibility to other stress factors. *Front. Plant Sci.* 8:1975. 10.3389/fpls.2017.01975 29201036 PMC5696719

[B93] Zhang Z. Z. Zhou J. Zhao X. H. Zhao P. Zhu L. W. Ouyang L. (2017b). Maximised photosynthetic capacity and decreased hydraulic failure risk during aging in the clump bamboo, Bambusa chungii. *Funct. Plant Biol.* 44 785–794. 10.1071/FP16381 32480607

