# Supplementary material for: Growing in Mixed Stands Increased Leaf Photosynthesis and Physiological Stress Resistance in Moso Bamboo and Mature Chinese Fir Plantations
Source: Front Plant Sci. 2021 May 20;12:649204. doi: 10.3389/fpls.2021.649204 (PMC8173113; doi:10.3389/fpls.2021.649204)
Supplement: Supplementary file 1 [file Table_1.doc]

**Growing in** **mixed stands increased leaf photosynthesis and** **physiological stress resistance in moso bamboo and mature Chinese fir plantations**

Chunju Peng^a, b, c, d^, Yandong Song^a, b, c, d, e^, Chong Li^a, b, c, d^, Tingting Mei^a, b, c, d^, Zhili Wu^a, b, c, d^, Yongjun Shi^a, b, c, d^, Yufeng Zhou^a, b, c, d^, Guomo Zhou^a, b, c, d, *^

*a State Key Laboratory of Subtropical Silviculture, Zhejiang A&F University, Lin'an 311300, Zhejiang, China.*

*b Zhejiang Provincial Collaborative Innovation Center for Bamboo Resources and High-efficiency Utilization, Zhejiang A&F University, Lin'an 311300, Zhejiang, China.*

*c Key Laboratory of Carbon Cycling in Forest Ecosystems and Carbon Sequestration of Zhejiang Province, Zhejiang A&F University, Lin'an 311300, Zhejiang, China.*

*d School of Environmental and Resources Science, Zhejiang A&F University, Lin'an 311300, Zhejiang, China.*

*e Lishui Academy of Agricultural and Forestry Sciences, Lishui 323000, Zhejiang, China*

*Corresponding author; Key Laboratory of Carbon Cycling in Forest Ecosystems and Carbon Sequestration of Zhejiang Province, Zhejiang A&F University, Lin'an 311300, Zhejiang, China.

E-mail address: zhougm@zafu.edu.cn (G. Zhou).

Running title: Mixing improved photosynthesis and resilience

**Table S1.** Parameter estimates for the selected fixed-effects model fitted to explain changes in on photosynthetic performance parameters (Amax, WUE, CUE, Fv/Fm, NPQ and chlorophyll content).

| species |  | Parameter | Estimate | sig |
| --- | --- | --- | --- | --- |
| one-year-old moso bamboo | Amax | mixture | 0.619 | *** |
|  |  | age | -1.267 | *** |
|  |  | species | -0.413 | *** |
|  | WUE | mixture | -1.103 | *** |
|  |  | age | -1.342 | *** |
|  |  | species | -0.406 | * |
|  | CUE | mixture | 0.005 | ** |
|  |  | age | -0.005 | *** |
|  |  | species | -0.001 |  |
|  | Fv/Fm | mixture | 0.015 | ** |
|  |  | age | 0.026 | *** |
|  |  | species | 0.066 | *** |
|  | NPQ | mixture | 0.217 | *** |
|  |  | age | 0.054 |  |
|  |  | species | 0.011 |  |
|  | chlorophyll content | mixture | 0.946 |  |
|  |  | age | 0.822 |  |
|  |  | species | 21.393 | *** |
| two-year-old moso bamboo | Amax | mixture | 0.329 | *** |
|  |  | species | 0.855 | ** |
|  | WUE | mixture | -0.094 |  |
|  |  | species | 0.935 | *** |
|  | CUE | mixture | 0.001 | * |
|  |  | species | 0.004 | *** |
|  | Fv/Fm | mixture | 0.024 | *** |
|  |  | species | 0.040 | *** |
|  | NPQ | mixture | -0.142 | *** |
|  |  | species | -0.043 |  |
|  | chlorophyll content | mixture | 1.312 |  |
|  |  | species | 20.571 | *** |
| Chinese fir | Amax | mixture | 0.373 | *** |
|  | WUE | mixture | 4.019 | ** |
|  | CUE | mixture | 0.008 | *** |
|  | Fv/Fm | mixture | 0.017 | *** |
|  | NPQ | mixture | -0.108 | * |
|  | chlorophyll content | mixture | 4.231 | * |

* *p* < 0.05; * **p* < 0.01; *** *p* < 0.001.
